# The Role of Surgery, Radiosurgery and Whole Brain Radiation Therapy in the Management of Patients with Metastatic Brain Tumors

**DOI:** 10.1155/2012/952345

**Published:** 2011-10-16

**Authors:** Thomas L. Ellis, Matthew T. Neal, Michael D. Chan

**Affiliations:** ^1^Department of Neurosurgery, Wake Forest University School of Medicine, Winston-Salem, NC 27157, USA; ^2^Department of Radiation Oncology, Wake Forest University School of Medicine, Winston-Salem, NC 27157, USA

## Abstract

Brain tumors constitute the most common intracranial tumor. Management of brain metastases has become increasingly complex as patients with brain metastases are living longer and more treatment options develop. The goal of this paper is to review the role of stereotactic radiosurgery (SRS), whole brain radiation therapy (WBRT), and surgery, in isolation and in combination, in the contemporary treatment of brain metastases. Surgery and SRS both offer management options that may help to optimize therapy in selected patients. WBRT is another option but can lead to late toxicity and suboptimal local control in longer term survivors. Improved prognostic indices will be critical for selecting the best therapies. Further prospective trials are necessary to continue to elucidate factors that will help triage patients to the proper brain-directed therapy for their cancer.

## 1. Introduction

Brain metastases are the most common intracranial tumor, arising in 10%–40% of all cancer patients [1, 2] and accounting for up to 170,000 new cases per year in the United States [3]. The observation of rising incidence is most likely related to the aging population, improved systemic treatment for the primary disease, and improved imaging techniques [4]. As a result, brain metastases are an increasing source of morbidity and mortality as well as cognitive impairment at the time of cancer diagnosis [5, 6]. Cancers with a high incidence in the general population (e.g., lung and breast) are the most frequently encountered source of brain metastases, accounting for up to two thirds of new cases [7]. Solid tumors constitute 95% of brain metastases, while leptomeningeal involvement makes up the remaining 5% [8–10]. Approximately 50%–60% of patients with solid tumors present with multiple metastases, while the remaining patients harbor a single mass [1, 11, 12]. The prognosis for patients with brain metastases from any histology is poor overall, with a median survival of only 4–7 months following treatment with WBRT alone [12–22]. For patients harboring a single, surgically amenable lesion, resection followed by WBRT has been found to be favorable to WBRT alone in two of three randomized controlled trials [17, 19, 22]. The local control rates and overall survival for patients with a single metastasis treated either with surgical resection followed by WBRT or with stereotactic radiosurgery (SRS) alone have been found to be similar [18, 19, 22–28]. On the other hand, SRS may yield superior local control rates for radioresistant brain metastases (e.g., from melanoma and renal cell) and provides the ability to treat unresectable lesions. Recent series have documented an improving survival among patients with brain metastases, suggesting that improving systemic therapies may be prolonging the lives of patients with brain metastases [29]. Such improvements imply that brain metastasis-directed therapies may have increasing importance among longer-term survivors. The purpose of this paper is to review the role of SRS, WBRT, and surgery—either alone or in combination—in the contemporary treatment of brain metastases.

## 2. SRS plus WBRT versus WBRT Alone

Several series have showed improvement in local control with the addition of SRS to WBRT when compared to WBRT alone. However, the detection of an overall survival advantage has been elusive. This is likely because there are specific populations that may benefit from an SRS boost.

The first prospective trial looking at the utility of an SRS boost was published in 1999 by Kondziolka et al. This single-institution RCT included patients with 2–4 metastases measuring ≤2.5 cm and a KPS ≥70 [27]. There were 14 patients in the WBRT arm and 13 in the WBRT plus SRS arm. The two groups were well matched with regard to gender, age, number of lesions, KPS score, histology, and extent of extracranial disease. Although the overall followup was not reported, the study had the advantage of no crossover between groups. The primary endpoint was local control with secondary endpoints including time to progression and median survival. The study was halted at the 60% accrual point after interim evaluation revealed substantially improved median time to progression (36 versus 6 months) and local failure rate (8% versus 100%) for patients in the WBRT plus SRS arm. Because of the small number of patients, there was insufficient power to assess differences in median survival. 

In 2004, Andrews et al. reported the results of the Radiation Therapy Oncology Group (RTOG) multicenter trial examining the use of WBRT versus WBRT plus SRS [30]. This was a randomized controlled study involving patients with 1–3 brain metastases, a Karnofsky performance status (KPS) of ≥70, and a lesion size of <4 cm for the largest lesion. Patient groups were matched for age, gender, KPS, histology, and scores on minimental status exam (MMSE). They were stratified by the number of lesions and the extent of extracranial disease. The primary endpoint was median survival with secondary endpoints of tumor control at 1 year, KPS and MMSE at 6 months, and cause of death. There were 167 patients in the WBRT alone arm, 28 (17%) of whom crossed over to receive SRS for salvage. The WBRT plus SRS arm contained 164 patients, 31 (19%) of whom did not receive the planned SRS boost. The trial demonstrated significantly improved survival for patients with single metastatic lesions, better local control for patients with 1–3 lesions (*P* = 0.001), and improved KPS for patients with 1–3 lesions in the WBRT plus SRS arm. There was no statistically significant difference between groups in incidence of neurologic death, MMSE at 6 months, toxicity, or median survival in patients with 2-3 metastases. Criticisms of the trial include the absence of imaging followup on 43% of the patients, a large crossover rate between the arms, and inclusion of tumors >3 cm which are known to be less amenable to SRS. 

Li et al. in 2000 reported their results of a three arm prospective cohort study examining patients with either small-cell or nonsmall-cell lung cancer with a single brain metastasis measuring ≤4.5 cm [31]. Seventy lung cancer patients with newly diagnosed single brain metastasis were treated with either WBRT alone (*n* = 29), or SRS alone (*n* = 23), or the combination of both (*n* = 18). Groups were well matched with regard to gender, age, extent of extracranial disease, histology, and KPS scores. Multiple endpoints, including survival, freedom from local progression, freedom from new brain metastasis, local control, KPS, and cause of death, were measured and compared using univariate and multivariate analyses. Analysis revealed improved median survival (*P* < 0.0001), local control (*P* = 0.004), and median time to progression (*P* < 0.0001) in the WBRT plus SRS arm. However, the comparison between SRS alone and SRS plus WBRT groups indicated that adding WBRT only improved freedom from distant failure. 

A large retrospective cohort trial performed by Sanghavi et al. evaluated 502 patients from 10 institutions with brain metastases from varying histologies [32]. They compared this group to historical controls based on recursive partitioning analysis (RPA) of a database from RTOG trials containing 1200 patients who received WBRT alone. The patients were stratified by RPA class (number of brain mets, presence of systemic disease, and KPS score) and were similar in gender, age, and extent of extracranial disease. The authors observed statistically significant improvements in survival for patients receiving WBRT plus SRS regardless of RPA class. One criticism of the study is that the WBRT plus SRS group had a larger percentage of radioresistant histologies and the WBRT group had slightly lower initial KPS scores.

On the basis of the two randomized controlled trials above, there is class I evidence that SRS plus WBRT yields significantly improved local tumor control rates compared to WBRT alone in patients with 1–3 brain metastases. Moreover, the study by Andrews et al. [30] suggests that there are particular populations such as RPA class I patients and those with single brain metastases that have an overall survival benefit with SRS boost. It is likely that the patients who benefit from an SRS boost are the ones that have an improved life expectancy, as these patients will live long enough to potentially experience the sequelae of local brain failure. 

## 3. SRS plus WBRT versus SRS Alone 

The late toxicities of WBRT have been well documented. Moreover, patients with longer survival times ultimately have an increased chance of experiencing WBRT-related toxicity [33]. As such, an approach of eliminating or delaying WBRT in patients with brain metastases would potentially improve cognitive outcomes in these patients. Multiple randomized trials have now been published showing no decrease in overall survival in patients in whom SRS alone was used to treat ≤4 brain metastases. 

A randomized controlled trial evaluating these two treatments was performed by Aoyama et al. and published in 2006 [34]. Patients with up to four metastases ≤3 cm and a KPS of ≥70 were randomized to either SRS alone (*n* = 67) or SRS plus WBRT (*n* = 65). The primary endpoint was median survival while secondary endpoints included distant failure, local control, and KPS scores at one year. Additional secondary endpoints included incidence of neurologic death, MMSE scores, and adverse events. Those patients receiving the combined treatment had the SRS dose reduced by 30%. The two patient groups were well matched with regard to gender, age, number of lesions, histology, extent of extracranial disease, and KPS scores. The follow-up period was 21 months for the SRS arm and 31 months for the SRS plus WBRT arm. Of the 67 patients in the SRS arm, 11 (16%) required WBRT for salvage. In the SRS plus WBRT arm, 6 (9%) of patients did not receive SRS, 2 (3%) did not receive WBRT, and 10 (15%) received additional SRS for salvage. Analysis revealed no significant difference between the two groups with regard to median survival, local control rate, KPS score, incidence of neurologic death, MMSE score, or adverse events. However, the SRS arm had a significantly higher likelihood local and distant failure at one year requiring salvage treatment with either additional SRS or WBRT. 

In a recent phase III trial, Kocher et al. compared patients with 1–3 brain metastases, excluding small-cell lung cancer, and stable extracranial disease that were randomized to an observational cohort or WBRT cohort following initial treatment with either SRS or surgery. Of the 199 patients in the radiosurgery group, 100 patients were allocated to observation, and 99 were allocated to WBRT. Among patients treated with surgery or SRS initially, there was no significant difference (*P* = 0.71) in the time to performance status decline for the observation group (10 months) versus the WBRT group (9.5 months). Similarly, overall survival times were not significantly different (*P* = 0.89) between the observation and WBRT arms (10.9 versus 10.7, resp.). They concluded adjuvant WBRT reduces intracranial relapses and neurologic deaths but fails to improve the duration of functional independence and overall survival after SRS or surgical treatment of cerebral metastases [35]. 

Chang et al. reported their results in 2009 of 58 patients with 1–3 metastases who were randomized either to SRS alone (*n* = 30) or SRS plus WBRT (*n* = 28) [36]. The primary endpoint was cognitive status at 4 months, while secondary endpoints were local control and overall survival. Although the overall survival was higher in the SRS group (15.2 versus 5.7 months), the 1-year local and distant failure rates were higher. Six percent of the patients in the SRS plus WBRT group required salvage SRS, while 33% of the patients in the SRS group required salvage resection and WBRT. Verbal memory testing revealed significantly less cognitive decline in the SRS group. 

There are multiple retrospective cohort studies (class II evidence) that reveal similar survival results in patients receiving SRS alone versus those receiving SRS plus WBRT [37–44]. These studies contain conflicting evidence, however, regarding the risk of local recurrence in the patients receiving SRS alone. As a result, while SRS alone is a reasonable treatment option in patients with up to 4 brain metastases, it should be followed by frequent MRI surveillance for local and distant failure. 

## 4. SRS Alone versus WBRT

At this time, there are no randomized trials directly comparing these two treatment modalities. In the three-armed trial by Li et al. alluded to above, patients treated with SRS alone demonstrated significantly longer median survival time (9.3 months) compared to those receiving WBRT alone (5.7 months) [31]. In three other studies (class II), there was a statistically significant survival advantage for patients receiving SRS alone for either single or multiple tumors compared to those receiving WBRT [45–47]. One class III study revealed similar outcomes for either treatment modality [48], while a second showed a significant survival advantage for SRS alone [49]. On the basis of this data, SRS alone is a reasonable treatment option for patients with limited brain disease although concern about distant failure necessitates frequent radiographic followup. 

## 5. Surgery plus WBRT versus WBRT Alone

WBRT has been the mainstay treatment for brain metastases for many years and continues to play a vital role in the management of patients with this disease. However, combining WBRT with surgical resection of symptomatic, accessible lesions can improve survival and quality of life. In a randomized trial (class I) performed by Patchell et al., surgical resection followed by WBRT (*n* = 25) was compared to WBRT alone (*n* = 23) for patients with a single metastatic brain tumor [19]. The trial demonstrated significantly improved survival in the surgery plus WBRT arm (40 weeks) compared to the WBRT alone group (15 weeks). 

In a second randomized trial (class I) involving 63 patients by Vecht et al., the outcome after surgical excision of a single brain metastasis followed by WBRT was compared to that after WBRT alone [22]. WBRT was delivered in a novel scheme of 2 fractions of 2 Gy per day for a total of 40 Gy. Patients were stratified by primary site (lung cancer versus nonlung cancer) and status of extracranial disease (progressive versus stable). Survival and functionally independent survival (FIS) were compared between the treatment arms. The arm undergoing combined treatment had a longer survival (*P* = 0.04) and longer FIS (*P* = 0.06) compared with the group receiving radiotherapy alone. The findings were most pronounced in patients with stable extracranial disease (median survival, 12 versus 7 mo; median FIS, 9 versus 4 mo). Patients with progressive extracranial disease had a median survival of 5 months and an FIS of 2.5 months regardless of the treatment received. 

The third, and largest, randomized trial (class I) evaluating these two treatment modalities was subsequently performed by Mintz et al. in 1996 [17]. Forty-three patients received radiation alone (3000 cGy in 10 fractions), while 41 patients underwent surgery plus radiation. The median survival for the WBRT alone group was 6.3 months compared with 5.6 months for the surgery plus WBRT group (*P* = 0.24). The majority of patients died within the first year (70% in the WBRT alone arm versus 88% in the surgery plus WBRT arm). The authors concluded that the trial failed to demonstrate that the addition of surgery to radiation therapy improved outcome of patients with a single brain metastasis. It should be noted that the study may have failed to show a difference in treatment regimens, because the study included patients with low KPS and extensive systemic disease. 

A recent, randomized trial by Kocher et al. compared patients with up to 3 brain metastases and stable extracranial disease that were randomized to an observational cohort or WBRT cohort following initial treatment with either SRS or surgery. There were 359 total patients in the study, 160 of whom received surgery. Although the study did not directly compare patients receiving surgery plus WBRT to patients receiving WBRT alone, they reported no advantage to adjuvant WBRT after surgery compared to surgery or SRS followed by observation in terms of the time to performance status decline (9.5 versus 10.0 months, resp., *P* = 0.71) or overall survival times (10.7 versus 10.9 months, resp., *P* = 0.89). They concluded adjuvant WBRT reduces intracranial relapses and neurologic deaths but fails to improve the duration of functional independence and overall survival after SRS or surgical treatment of cerebral metastases [35]. 

Two of these three randomized controlled trials demonstrate that the addition of resection to WBRT rather than WBRT alone leads to improved survival and functional status in patients with a single, surgically accessible brain metastasis. A fourth study found no survival benefit to adding adjuvant WBRT to surgical resection. While much of this data was acquired twenty years ago, it continues to be quite relevant in the management brain metastases. It should be recognized, however, that the majority of patients enrolled in these trials were likely symptomatic or had larger metastases, as these were the common presentation of brain metastases in that era. 

## 6. Surgery Plus WBRT versus Surgery Plus SRS

At this time, there are no prospective studies comparing these two treatment modalities. In a retrospective cohort study by Serizawa et al. of patients with multiple metastases from non-small cell lung cancer, 34 patients received resection followed by WBRT while 62 underwent SRS [50]. Of the latter group, 23 were treated with SRS either before or after resection of a lesion >3 cm. The patients who received SRS demonstrated significantly longer survival time (12.5 versus 6.6 months). The survival time of the subpopulation of 23 patients who underwent resection in combination with SRS was not reported. The median time to distant brain failure was 14 months in the SRS group and 17.6 months in the group receiving WBRT although the difference was not statistically significant. 

Given the paucity of studies comparing these two treatment strategies, there is insufficient evidence to justify one over the other. However, the CALGB is in the process of designing a prospective randomized trial that will randomize patients with resected brain metastases to adjuvant WBRT versus cavity-directed SRS plus SRS to any remaining metastases. It is likely that this study will begin accrual in 2012.

## 7. Surgery Plus WBRT versus SRS Plus WBRT

The major issue underlying these two techniques is optimization of local control in cases where single modality therapy is likely insufficient. Larger brain metastases generally have a higher local failure rate after surgery or SRS alone. As such combined modality therapy offers the ability to add a second modality to improve local control. At this time, there are no prospective studies comparing these two treatment modalities although there are four retrospective studies. Bindal et al. reported their results in 1996 comparing 62 patients with a single brain metastasis (<3 cm) treated with resection ± WBRT to 31 patients who underwent SRS ± WBRT [51]. Patients were well matched with regard to gender, age, KPS, extent of resection, and histology. WBRT was actually completed by 66% of the patients in the first arm and 71% of patients in the second arm. The authors observed that those patients in the first arm (resection plus WBRT) demonstrated significantly longer median time to recurrence and median survival (16.4 versus 7.5 months) compared to the second arm. In addition, those patients in the first arm had a significantly lower rate of neurologic death and adverse events related to treatment. 

O'Neill et al. reported their retrospective analysis of 97 patients with solitary brain metastases, 74 of whom underwent resection, while the remaining patients received radiosurgery [52]. The use of WBRT was similar between the two groups (82% of surgical patients and 96% of radiosurgery patients). While there was a trend toward increased 1-year survival in the patients who underwent resection (62% versus 56%), this did not reach statistical significance. However, there was a significant increase in local tumor control for the patients receiving radiosurgery (100% versus 42%) at a median followup of 20 months.

By contrast Schöggl et al. found improved results in patients receiving radiosurgery rather than resection in a retrospective, case-control study comparing these treatment modalities [53]. They followed 133 patients whose treatment for a single brain metastasis was either radiosurgery or surgical resection. Only patients who received additional WBRT were included in the study. Sixty-seven patients were treated by radiosurgery and 66 patients were treated by resection. Standard radiosurgical and WBRT doses were used. The median survival for patients receiving radiosurgery was 12 months compared to 9 months for patients after resection, a difference that did not reach statistical significance. There was, however, a significant difference in local control favoring the patients undergoing radiosurgery (95 versus 83%, *P* < 0.05). 

Another recent, randomized trial by Kocher et al. compared patients with up to 3 brain metastases and stable extracranial disease that were randomized to an observational cohort or WBRT cohort following initial treatment with either SRS or surgery. While the study did not directly compare patients receiving patients receiving surgery plus WBRT to patients receiving SRS plus WBRT, they found no advantage to adding WBRT to either of the initial treatment modalities in terms of the time to performance status decline (10 versus 9.5 months, resp., *P* = 0.71) or overall survival times (10.9 versus 10.7, resp., *P* = 0.89) [35]. 

While the first two studies above reveal statistically significantly longer survival in patients receiving resection, the third study demonstrated a trend towards longer survival in those receiving SRS although it did not reach statistical significance. Further, Kocher's study did not reveal an advantage with adjuvant WBRT for prolonged survival or performance status after surgery or SRS. Both the Joint Center for Radiation Therapy and MD Anderson attempted randomized phase III trials comparing primary radiosurgery to surgery in the 1990s. Neither of these studies were able to accrue sufficient patients for analysis because of physician bias. As a result, both strategies are reasonable in patients with solid tumors <3 cm in diameter. However, surgery may be more suitable for patients with symptomatic, accessible lesions in order to palliate symptoms. 

## 8. Surgery plus WBRT versus SRS Alone

There is one randomized controlled trial (class I) and two retrospective cohort studies (class II) comparing these two modalities. Muacevic et al. performed a multicenter prospective randomized trial involving 33 patients treated with resection followed by WBRT and 31 patients treated with SRS alone [54]. All tumors were ≤3 cm in maximum diameter, and all patients had a KPS ≥70. The primary endpoint of the study was overall survival with secondary endpoints of local control, toxicity, and quality-of-life measures. Treatment results did not differ in terms of survival (*P* = 0.8), neurological death rates (*P* = 0.3), and freedom from local recurrence (*P* = 0.06). Patients in the radiosurgery group were more likely to demonstrate distant failure (*P* = 0.04). Radiosurgery was associated with a lower frequency of adverse events (*P* < 0.01). Improved scores for quality of life measures were seen 6 weeks after radiosurgery (*P* < 0.05). It should be noted that quality of life measures were no different in the radiosurgery and surgery groups at 6 months after treatment. The authors concluded that the main advantage of radiosurgery remains its less invasiveness in terms of hospital stay, duration of treatment, and risk of short-term toxicities. 

The same group previously performed a retrospective cohort evaluation of 56 patients who underwent radiosurgery alone versus 52 patients treated with resection followed by WBRT [55]. The tumors were ≤3.5 cm and the two groups were well matched with regard to gender, age, KPS scores, and extent of extracranial disease. The 1-year survival rate was 53% (68 weeks) in the surgical group and 43% (35 weeks) in the radiosurgical group (*P* = 0.19). The 1-year rate of local control after surgery and radiosurgery were 75% and 83%, respectively (*P* = 0.49). The 1-year rate of neurological death was 37% in the surgical group and 39% in the radiosurgical group (*P* = 0.8). A pretreatment KPS score <70 was predictive of worsened survival. Periprocedural morbidity and mortality rates were 7.7% and 1.6% in the resection group and 8.9% and 1.2% in the radiosurgery group. 

Shinoura et al. performed a retrospective cohort evaluation of 28 patients who underwent radiosurgery alone compared to 35 patients treated with resection and radiotherapy [56]. The two groups were well matched with regard to gender, age, and histology but the extent of systemic disease was not reported. Moreover, some of the patients received local RT as opposed to WBRT, and the number of these patients was not reported. Nonetheless, the authors reported significantly longer median survival (34.4 versus 8.2 months) in the surgery plus RT arm. This arm also demonstrated longer mean time to recurrence (25 versus 7.2 months) during the follow-up period. 

Given the inconclusive results of these studies, no definitive recommendations can be made regarding the superiority of one of these treatment strategies over the other. 

## 9. Surgery plus SRS versus SRS Alone

There are no studies comparing these two treatment options. In general, surgery tends to be useful in larger lesions. Multiple studies have reported good local control rates with radiosurgical treatment of a resection cavity [34, 57–62]. Do et al. described their results with 30 patients with brain metastases treated with resection followed by SRS or stereotactic radiotherapy (4–6 fractions) to the resection cavity [57]. Of the 30 patients, 4 (13.3%) developed recurrence in the resection cavity, and 19 (63%) developed distant brain failure. The actuarial 12-month survival rates were 82% for local recurrence-free survival, 31% for freedom from new distant brain metastases, 67% for neurologic deficit-free survival, and 51% for overall survival. Salvage WBRT was performed in 14 (47%) of the 30 patients. 

Jagannathan et al. reported their results with 47 patients who underwent SRS to the postoperative resection cavity following resection of a brain metastasis [59]. In addition to the resection cavity, 34 patients (72%) underwent SRS to 116 intact metastases. The radiographic end point was defined as local tumor control. The mean volume of the treated cavity was 10.5 cm^3^ (range 1.75–35.45 cm^3^), and the mean marginal dose to the cavity was 19 Gy. Clinical end points were defined as KPS and survival. The mean duration between resection and SRS was 15 days. During a mean follow-up duration of 14 months, local tumor control at the site of the surgical cavity was achieved in 44 patients (94%). Recurrence in the cavity was statistically related to the volume treated (*P* = 0.04). At the last followup evaluation, the mean KPS score for the group was 78 (median 80, range 40–100). During the follow-up period, 34 patients (72%) underwent additional SRS for new metastases. At the time of last follow-up, 11 patients were alive and 36 patients had died (mean duration until death 12 months). Patients with good control of their primary tumor had improved survival compared to those who did not (*P* = 0.004). 

A 2009 study by Karlovits et al. involved a retrospective review of 52 patients with 1–4 brain metastases treated at their institution with SRS to a resection cavity [60]. A single metastasis was resected in each patient. A median dose of 1500 cGy (range 800–1800 cGy) was delivered to the margin of the resection cavity. The median cavity volume was 3.85 cm^3^ (range 0.08–22 cm^3^). With a median follow-up duration of 13 months, the median survival was 15.0 months. Four patients (7.7%) developed local recurrence within the resection cavity. Twenty-three patients (44%) developed distant brain recurrences at a median of 16 months after resection. Sixteen (30.7%) received salvage WBRT. The median time to WBRT delivery was 8.7 months (range 2–43 months). Multivariate analysis revealed a significantly longer survival for patients with no extracranial disease on presentation (*P* = 0.01) and solitary brain metastasis (*P* = 0.02). No factor (age, RPA class, tumor size or histological type, extent of extracranial disease, extent of resection, or SRS dose or volume) was related to the need for salvage WBRT. 

Hwang et al. analyzed their experience delivering SRS to the tumor cavity following surgical resection of brain metastases and compared their results to patients receiving WBRT after surgical resection [58]. Twenty-five patients had a metastatic lesion resected followed by adjuvant GKS to the resection cavity. The median survival for patients receiving SRS to the resection cavity was 15 months as compared to 6.8 months for those receiving WBRT (*P* = 0.08). 

Jensen et al. recently described our experience at Wake Forest University with 106 patients with no prior WBRT who were treated using radiosurgery directed to the tumor cavity and to any synchronous brain metastases detected at the time of SRS planning [29]. A median dose of 17 Gy to the 50% isodose line was prescribed to the margin of the resection cavity. Patients were followed up via serial imaging, and new brain metastases were generally treated using additional SRS, with salvage WBRT typically reserved for local treatment failure at a resection cavity, numerous failures, or failures occurring at short time intervals. SRS was delivered to the resection cavity alone in 57.5% of patients, whereas 24.5% of patients also received treatment for 1 synchronous metastasis, 11.3% for 2 synchronous metastases, and 6.6% for 3–10 additional lesions. Overall survival at 1 year was 46.8%. The local tumor control rate at 1 year was 80.3%. The disease control rate in distant regions of the brain at 1 year was 35.4%, with a median time of 6.9 months to distant failure. Thirty-nine of 106 patients eventually required salvage WBRT, and the median time to salvage WBRT was 12.6 months. Kaplan-Meier estimates showed that the rate of requisite WBRT at 1 year was 45.9%. Neurological cause-specific survival at 1 year was 50.1%. Leptomeningeal failure occurred in 8 patients. Seven patients required repeat surgery. Multivariate analysis revealed that preoperative tumor diameter >3 cm was predictive of treatment failure. 

Given the absence of comparative studies regarding these two treatment modalities, no recommendation can be made regarding the superiority of one over the other. However, SRS is inherently limited by the ability to control larger lesions (>2 cm) because of the inability to deliver adequate dose. Surgery with such larger lesions provides the advantage of potentially rendering the patient with only microscopic residual disease. Adjuvant radiosurgery in this setting likely has a better chance of controlling disease than it would with an intact tumor.

## 10. Discussion

As evidence has become available that patients with brain metastases are living longer than in the past [63, 64], the management of patients with brain metastases has become more complex. The complexity of this decision tree is underscored by the 2010 statement from the American College of Radiology's (ACR) Appropriateness Panel on single brain metastasis that indicates that there is no clear consensus regarding optimal or ideal treatment for these patients [65]. Table 1 demonstrates the major, prospective, randomized trials that represent the basis for current management strategies. 

While local control of brain metastases continues to be important, such issues as the long-term toxicity of treatment, and the optimal timing of WBRT are becoming equally important. Moreover, the indications for radiosurgical management of brain metastases continues to evolve. At the present time, the indications for radiosurgery include (1) boost after WBRT for patients with <5 brain metastases, (2) as monotherapy to avoid the cognitive toxicities of WBRT in patients with <5 brain metastases, (3) as salvage therapy after failure of previous WBRT, (4) radioresistant brain metastases (e.g., renal cell carcinoma, melanoma, and sarcoma), and (5) adjuvant therapy for a resected radioresistant brain metastases (e.g., renal cell carcinoma, melanoma, and sarcoma). For patients receiving SRS as a boost after WBRT, this is generally reserved for patients with potentially longer life expectancies who received WBRT. The previous randomized trial has suggested that patients in the better prognostic group (e.g., 1 metastasis, or no extracranial disease), tended to have improved survival with radiosurgical boost. This is likely because patients with a greater life expectancy have a longer potential time during which to experience a local failure. 

Two published randomized trials have now demonstrated that treating patients with <4 metastases with radiosurgery alone not only does not lead to worsened overall survival, but may also improve neurocognitive outcomes. These data are somewhat controversial, however, given previous dogma that distant or local brain failures are among the prominent factors leading to neurocognitive decline in this patient population. Furthermore, local failure after radiosurgery appears to be a time-dependent phenomenon, and late local failures appear to be more common in patients who receive radiosurgery alone as compared to WBRT with a radiosurgical boost. 

Perhaps more than ever, the use of prognostic indices to triage patients with brain metastases into the proper treatment algorithm is paramount. The RTOG published a recursive partitioning analysis of patients with brain metastases, separating patients based on 3 prognostic groups, and basing these groupings on such variables as age, KPS, and status of extracranial disease [26]. However, these data were collected several decades ago, and the improvement in systemic therapies and lead-time biases created by improved imaging and surveillance may have caused this prognostic index to become obsolete. Further efforts to establish a more modern prognostic index has given rise to the graded prognostic assessment (GPA) based on histology-specific prognostic factors [66]. Prognostic indices, while imperfect, allow the treating physician to estimate the life expectancy of the patient, and subsequently determine if WBRT can be delayed or given upfront. Future prognostic indices may ultimately incorporate failure patterns after SRS alone, in order to help determine the patients who will fail outside of the SRS treatment volume early on, and who may potentially benefit from upfront WBRT.

Perhaps one of the most important indications for SRS is salvage of WBRT failures. The alternative to SRS in the setting of WBRT failure is repeat WBRT. This option is significantly limited by toxicity and the ability to deliver sufficient doses to the brain metastases. Several retrospective series have documented median survival times of approximately 8 months after radiosurgical salvage of WBRT failures. Chao et al. found that patients with brain metastases who recurred after WBRT and were treated with salvage SRS had good local control and survival rates after SRS [67]. Those patients with a longer time to failure after WBRT had significantly longer survival after SRS. Two other large retrospective studies have demonstrated that SRS as a salvage treatment following initial SRS can provide excellent local tumor control in patients with 4 or fewer metastases [68, 69]. 

SRS has also been widely used as an initial monotherapy for radioresistant brain metastases such as renal cell carcinoma, melanoma, and sarcoma. The seminal series demonstrating suboptimal outcomes with WBRT alone for such radioresistant tumors was published by investigators at MD Anderson showing a rate of neurologic death as high as 76% in patients with renal cell carcinoma receiving WBRT alone [70]. On the other hand, several series have described a high rate of durable location control and much lower rates of neurologic death in series of patients receiving radiosurgical management [71]. Clarke et al. have examined the outcomes of patients with a single brain metastasis from radioresistant histologies (renal cell carcinoma and melanoma) treated with stereotactic radiosurgery (SRS) [72]. On the Basis of their small retrospective series, they concluded SRS is a safe and feasible strategy for treatment of patients with a limited number of radioresistant brain metastasis. In those few patients who received WBRT, local control, progression-free survival, and overall survival were not significantly improved. 

The role of surgical resection for brain metastases has been well established. Large or symptomatic lesions will commonly benefit from the decompressive effects of surgery. In addition, SRS is inherently limited by the ability to deliver sufficient tumoricidal doses to lesions greater than 2 cm. In these scenarios, surgery will provide improved local control without the risk of radionecrosis. The surgical literature should be interpreted in terms of the historical context that most metastases detected during the era in which these trials were conducted were either symptomatic or diagnosed on CT. As such, there was probably a bias towards larger and symptomatic lesions. Nonetheless, two randomized controlled trials have demonstrated that the addition of surgical resection to WBRT rather than WBRT alone leads to improved survival and functional status in patients with a single, surgically accessible brain metastasis [19, 22]. As a result, it is general practice that all metastases larger than 3 cm in surgically accessible regions should receive serious consideration for resection. 

The proper adjuvant therapy after surgical resection has yet to be determined. While several randomized trials have shown at least a local control benefit to adjuvant WBRT after resection of a brain metastasis, most have not shown a survival advantage. However, observation after surgical resection of a metastasis leads to a local failure rate of 50% within the resection cavity. A number of recent series have suggested that cavity-directed SRS as adjuvant therapy after surgery is a viable option. This technique allows for the delay or elimination of WBRT in most patients, while potentially delaying or even avoiding the toxicities of WBRT. The combined literature suggests a local control rate of approximately 80% at 1 year with cavity-directed SRS [29]. Improved imaging techniques allow for detection and treatment of synchronous brain metastases detected at time of SRS [73]. 

In conclusion, management of brain metastases continues to evolve over time On the basis of improving survivals of patients and improving methods of prognostication. Surgery and radiosurgery both offer management options that may help to optimize therapy in selected patients. WBRT is another option but can lead to late toxicity and suboptimal local control in longer term survivors. Further prospective trials are necessary to continue to elucidate factors that will help triage patients to the proper brain-directed therapy for their cancer.

## Figures and Tables

**Table 1:** Randomized Trials for Treatment of Brain Metastases.

Author	Treatment	Local Control (1 yr)	Distant Brain Failure (1 yr)	Overall Survival
Patchell [19]	WBRT	48%	13%	9% (1 yr)
	Surgery plus WBRT	80%	20%	45%
Vecht [22]	WBRT	NA	NA	23% (1 yr)
	Surgery plus WBRT	NA	NA	50%
Mintz [17]	WBRT	NA	NA	17% (1 yr)
	Surgery plus WBRT	NA	NA	30%
Patchell et al. [18]	Surgery	54%	37%	43 wks
	Surgery plus WBRT	90%	14%	48 wks (median)
Andrews et al. [30]	WBRT	71%	33%	23% (1 yr)
	WBRT plus SRS	82%	27%	29%
Kocher et al. [35]	SRS	70%	44%	47% (1 yr)
	SRS plus WBRT	87%	28%	46%
Chang et al. [36]	SRS	67%	55%	60% (1 yr)
	SRS plus WBRT	100%	27%	21%
Aoyama et al. [34]	SRS	76%	63%	28% (1 yr)
	SRS plus WBRT	90%	42%	39%
